# A Proline Derivative-Enriched Fraction from *Sideroxylon obtusifolium* Protects the Hippocampus from Intracerebroventricular Pilocarpine-Induced Injury Associated with *Status Epilepticus* in Mice

**DOI:** 10.3390/ijms21114188

**Published:** 2020-06-11

**Authors:** Pedro Everson Alexandre de Aquino, Jéssica Rabelo Bezerra, Tyciane de Souza Nascimento, Juliete Tavares, Ítalo Rosal Lustosa, Adriano José Maia Chaves Filho, Melina Mottin, Danielle Macêdo Gaspar, Geanne Matos de Andrade, Kelly Rose Tavares Neves, Giuseppe Biagini, Edilberto Rocha Silveira, Glauce Socorro de Barros Viana

**Affiliations:** 1 Department of Physiology and Pharmacology, Federal University of Ceará, Fortaleza 60430-270, Brazil; pedroeverson.alexandre@gmail.com (P.E.A.d.A.); jrabelo.b@gmail.com (J.R.B.); tycianesouza13@gmail.com (T.d.S.N.); juliete.tavares@hotmail.com (J.T.); adrianoafilho@hotmail.com (A.J.M.C.F.); daniellesm2000@yahoo.com (D.M.G.); gmatos@ufc.br (G.M.d.A.); kelly.rose@hotmail.com (K.R.T.N.); 2 PhD Program in Clinical and Experimental Medicine, University of Modena and Reggio Emilia, 41121 Modena, Italy; italo.rosal@gmail.com; 3 Laboratory of Molecular Modeling and Drug Design, LabMol, Faculty of Pharmacy, Federal University of Goiás, Goiás 74605-050, Brazil; melinamottin@gmail.com; 4 Laboratory of Experimental Epileptology, Department of Biomedical Metabolic and Neural Sciences, University of Modena and Reggio Emilia, 41121 Modena, Italy; 5 Center for Neuroscience and Neurotechnology, University of Modena and Reggio Emilia, 41121 Modena, Italy; 6 Department of Organic and Inorganic Chemistry, Federal University of Ceará, Fortaleza 60455-970, Brazil; edil@ufc.br

**Keywords:** mesial temporal lobe epilepsy, pilocarpine, GABA transporter 1, glial fibrillary acidic protein, ionized calcium-binding adaptor molecule 1, *N*-methyl-(2S,4R)-trans-4-hydroxy-l-proline, neuroprotection

## Abstract

The *N*-methyl-(2S,4R)-trans-4-hydroxy-l-proline-enriched fraction (NMP) from *Sideroxylon obtusifolium* was evaluated as a neuroprotective agent in the intracerebroventricular (icv) pilocarpine (Pilo) model. To this aim, male mice were subdivided into sham (SO, vehicle), Pilo (300 µg/1 µL icv, followed by the vehicle *per os*, *po*) and NMP-treated groups (Pilo 300 µg/1 µL icv, followed by 100 or 200 mg/kg *po*). The treatments started one day after the Pilo injection and continued for 15 days. The effects of NMP were assessed by characterizing the preservation of cognitive function in both the Y-maze and object recognition tests. The hippocampal cell viability was evaluated by Nissl staining. Additional markers of damage were studied—the glial fibrillary acidic protein (GFAP) and the ionized calcium-binding adaptor molecule 1 (Iba-1) expression using, respectively, immunofluorescence and western blot analyses. We also performed molecular docking experiments revealing that NMP binds to the γ-aminobutyric acid (GABA) transporter 1 (GAT1). GAT1 expression in the hippocampus was also characterized. Pilo induced cognitive deficits, cell damage, increased GFAP, Iba-1, and GAT1 expression in the hippocampus. These alterations were prevented, especially by the higher NMP dose. These data highlight NMP as a promising candidate for the protection of the hippocampus, as shown by the icv Pilo model.

## 1. Introduction

Epilepsy affects approximately 65 million people around the world [1], and apart from cerebrovascular diseases, is the third common neurological disorder after dementias and headache [2]. Different types of epilepsy exist, with a broad phenotypic spectrum ranging from mild to disabling and life-threatening states. They share recurrent unprovoked or reflex seizures as the hallmark [3]. Mesial temporal lobe epilepsy (MTLE) is the most prevalent type of adult epilepsy [4] and is characterized by hippocampal sclerosis [5], a lesion that has been related to a triggering event such as febrile seizures in childhood, facilitated by a genetic susceptibility background [6]. Hippocampal sclerosis has been hypothesized to contribute to refractoriness to antiepileptic drugs (AEDs), a condition that characterizes most patients with MTLE [7] even when biasing factors such as poor adherence to treatment have been accounted for [8,9]. Indeed, the poor responsiveness to AEDs requires a major effort for the health care system in assisting patients and undoubtedly represents a social burden for difficulties encountered by patients to achieve life satisfaction [10]. For these challenging reasons, more effective therapeutic options are required to address MTLE.

Drug development must be guided by the underlying pathophysiology of the considered disease. In this regard, seizures have been related to the imbalance of neuronal excitation and inhibition determined, respectively, by glutamate and γ-aminobutyric acid (GABA). Accordingly, GABA enhancers, i.e., agents which either bind directly to and positively modulate the type A GABA (GABA_A_) receptor (such as benzodiazepines and allopregnanolone), or delay the GABA clearance (tiagabine and vigabatrin) exhibit antiepileptic activity in MTLE [11]. An interesting target is the GABA transporter 1 (GAT1), which is the main GABA sink mechanism. This protein is expressed by neurons and astrocytes throughout the whole brain. By taking up GABA from synaptic clefts, GAT1 contributes to limit the synaptic-mediated phasic inhibition but also accounts for the homeostasis of the interstitial GABA concentration, contributing to the control of extrasynaptic GABA_A_ receptor-mediated tonic inhibition. In this way, GAT1 modulates neuronal network excitability in states of basal, as well as enhanced synaptic activity. GAT1 was the first GABA transporter of a four-member family of neurotransmitter-Na^+^ symporters to be cloned and crystallized [12]. Tiagabine, a drug used for the treatment of MTLE, acts by binding and inhibiting the GAT1-mediated GABA uptake mechanism. This ultimately leads to increased concentrations of the endogenous ligand, enhancing GABA-mediated hyperpolarization, so producing beneficial effects.

Neuroinflammation has also been involved in the pathophysiology of MTLE. Both types of glial cells, microglia, and astrocytes participate in neuroinflammation, which is mainly contributed by leukocytes at least in the acute phase [13]. Microglia are the brain resident immune cells. They can be activated by many stimuli, ultimately implying brain tissue function. Activated microglia release various mediators of inflammation after trauma and seizures, including metalloproteinase-12, leading to neuron degeneration, as well as astrogliosis [14]. Thus, microglia take an active part in the process of neural tissue remodeling, known as epileptogenesis, which has been thought to turn the healthy brain tissue hyperexcitable.

Astrocytes have also been recognized to play a role in epileptogenesis and ictogenesis. Apart from the widely known functions such as neurotransmitter clearance, ionic balance and buffering, glycogen storing, and selective permeability from capillaries, they also express cytokine receptors and secret trophic factors. Astrocytes may secrete molecules able to enhance or suppress ongoing inflammatory processes during neurologic diseases, including epilepsy [15]. Early studies [16,17] have shown that a single electroconvulsive seizure is able to elicit a remarkable increase in glial fibrillary acidic protein (GFAP) expression. This represents an initial step in the astrocyte’s reaction leading to their hypertrophy, whose precise contribution to epileptogenesis has to be enlightened.

A well-known consequence of damage to the hippocampus and related structures, as found in MTLE, is that patients have a prevalence of neuropsychiatric comorbidities significantly higher than age- and sex-matched healthy people [18]. Among the comorbidities that strongly impact patient’s life quality in MTLE are (i) cognitive impairments, i.e., impairment of learning and memory related to injury in the hippocampus, dentate gyrus, and entorhinal cortex [19], and (ii) mood or affective disturbances, i.e., anxiety and depression related to amygdala pathology [18,20,21]. Further, during seizures, the mesial temporal lobe structures give origin to dyscognitive auras, as well as vegetative and fear-related symptoms, due to ictal activation of, respectively, perirhinal cortex and amygdala [22]. Behavioral and cognitive deficits have also been consistently reported in animal models of MTLE [23,24].

Innovative drugs for patients with MTLE should be able to address both the acute and chronic mechanisms responsible for the seizures, neurodegeneration, and comorbidities, as previously outlined. Molecules obtained from biological sources have yielded important prototypes for drug development [25,26]. On this background, the shrub *Sideroxylon obtusifolium*, which grows spontaneously in semi-arid regions from Northeastern Brazil, is widely used in local folk medicine due to its wound healing, antinociceptive, anti-inflammatory, and claimed anti-seizure properties [27,28]. As we recently characterized the anti-inflammatory properties of *N*-methyl-(2S,4R)-trans-4-hydroxy-l-proline (NMP) from *Sideroxylon obtusifolium*, the objectives of the present work were to study the effects of NMP on the behavioral and brain changes occurring after intracerebroventricular (icv) pilocarpine (Pilo)-induced *status epilepticus* (SE) and brain damage, by considering the expression of GAT1, neuroinflammation, and gliosis, as evaluated by Nissl staining, immunohistochemistry assays, and western blotting. Specifically, we considered four groups of mice subdivided into the (i) sham group, surgically operated but not exposed to seizures, and given vehicle (hereafter referred as SO), (ii) Pilo-damaged and vehicle-treated group, and finally, (iii) two groups of Pilo-damaged mice treated with the NMP (Pilo + NMP) fraction from the medicinal plant *Sideroxylon obtusifolium* at two different doses. In addition, molecular docking experiments for GAT1 were also carried out.

## 2. Results

### 2.1. Behavioral Tests

**Figure 1:**
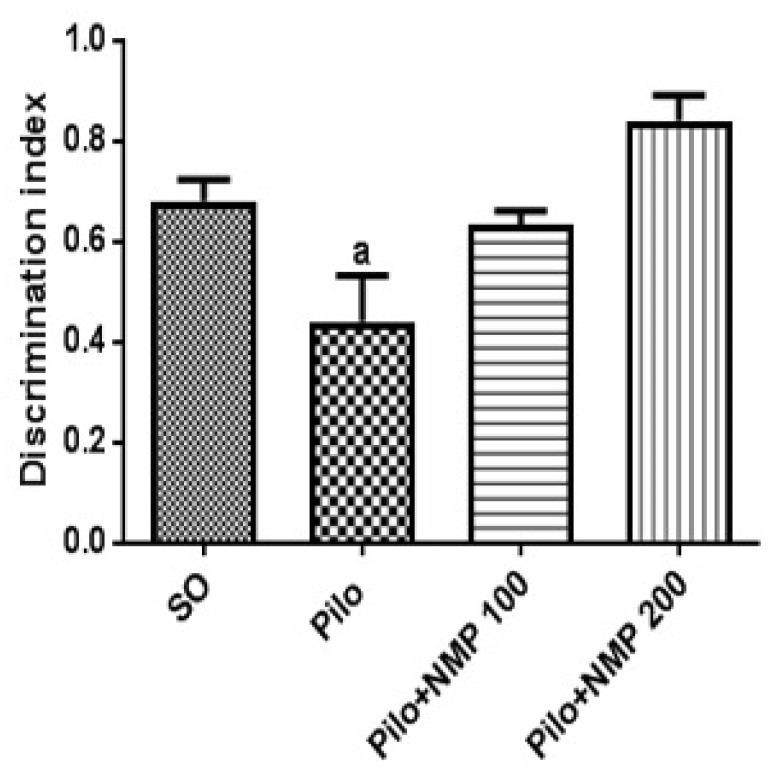
The *N*-methyl-(2S,4R)-trans-4-hydroxy-l-proline-rich fraction (NMP) prevented the behavioral changes in the recognition memory induced by the intracerebroventricular (icv) injection of pilocarpine (Pilo), evaluated by the novel object recognition test in mice. The animals received Pilo (300 µg/1 µL, icv) and, 24 h after, were treated for 15 days with NMP (100 or 200 mg/kg, *per os*, *po*; Pilo + NMP groups) or with distilled water (vehicle *po*; sham-operated, SO group). Groups were composed of eight animals/group, average). The behavioral tests were performed 1 h after the last drug administration. a. vs. SO, *p* < 0.0001 (one-way analysis of variance followed by Dunnett’s multiple comparisons test).

Three out of 27 mice died after Pilo treatment (11% mortality), whereas all SO mice survived. In the novel object recognition test (ORT) for short-term memory, control mice of the Pilo group exhibited significantly impaired performance compared to the SO group (Figure 1). On the other hand, treatment with *per os* (*po*) NMP at both doses of 100 mg/kg and 200 mg/kg was able to prevent the short term memory impairment related to pilocarpine-induced injury (*p* < 0.0001, one-way analysis of variance—ANOVA—followed by Dunnett’s test), since the performance of these NMP-treated animals was not different from the SO group.

**Figure 2:**
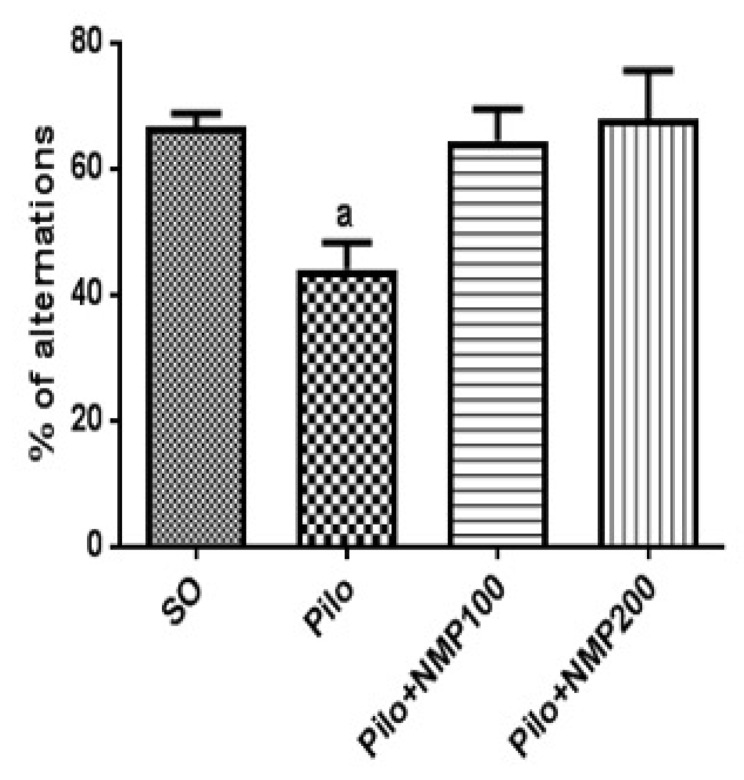
The *N*-methyl-(2S,4R)-trans-4-hydroxy-l-proline-rich fraction (NMP) prevented the behavioral changes in the operational memory, induced by the intracerebroventricular (icv) injection of pilocarpine (Pilo), evaluated by the Y-maze test in mice. The animals received Pilo (300 µg/1 µL, icv) and, 24 h after, were treated for 15 days with NMP (100 or 200 mg/kg, *per os*, *po*; Pilo + NMP groups) or with distilled water (vehicle *po*; sham-operated, SO). Groups were composed of eight animals/group on average. The behavioral tests were performed 1 h after the last drug administration. a. vs. SO, *p* = 0.0063 (one-way analysis of variance followed by Dunnett’s multiple comparisons test).

We also investigated the behavioral performance in the Y maze test, which was used to evaluate both short-term and operative memory. In this test, the Pilo group exhibited a significantly reduced percentage of spontaneous side alternation compared to the SO group (*p* = 0.0063, one-way ANOVA followed by Dunnett’s test, Figure 2). Treatment with NMP prevented the cognitive impairment significantly at both doses (Figure 2), thus preserving mouse memory functioning.

Even though we did not monitor the animals in the long-term, in order to record the occurrence of spontaneous recurrent seizures, which mainly appear from the 2nd week after Pilo injection onwards, it is worth to mention that we noticed an overt behavior consisting of remarkable aggressiveness and hyperreactivity in response to noises and handling. This behavior is often reported in animals with spontaneously recurrent seizures documented by electrocorticographic video recording in the Pilo MTLE model [29].

### 2.2. Nissl Staining and Neuronal Viability

**Figure 3:**
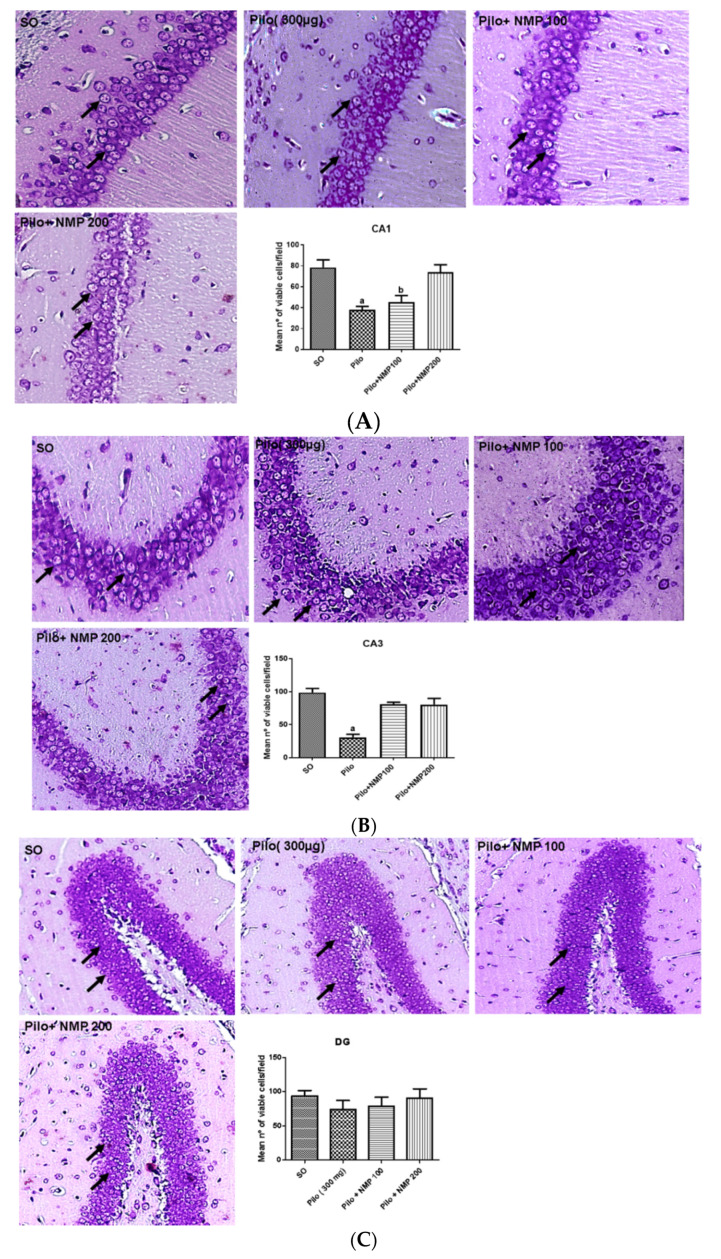
Representative photomicrographs (100 magnification) of the hippocampal *cornu Ammonis* (CA) subfields CA1 (**A**), CA3 (**B**), and (**C**) dentate gyrus (DG), showing that the *N*-methyl-(2S,4R)-trans-4-hydroxy-l-proline-rich fraction (NMP) increased the number of hippocampal viable cells in mice subjected to the intracerebroventricular (icv) injection of pilocarpine (Pilo). The animals received Pilo (300 µg/1 µL, icv) and, 24 h after, were treated with NMP (100 or 200 mg/kg *per os*, *po*), for 15 days. The sham-operated (SO) group received the vehicle, distilled water *po*. After the behavioral tests, animals were euthanized for histological analysis with cresyl violet (*n* = 4 animals/group, three slices/animal). Bar charts show the mean value of viable cells/field (seven fields/group). CA1: a. vs. SO, *p* < 0.0004; b. vs. SO, *p* < 0.001. CA3: a. vs. SO, *p* < 0.0001 (one-way analysis of variance, followed by the Dunnett’s test for multiple comparisons). Black arrows point to viable neurons. The cells were considered viable neurons when they presented violet staining in the cytoplasm, as well as normal morphological aspects (round or oval cells with centralized nuclei).

The Pilo treatment induced a remarkable reduction in Nissl stained neurons of the *cornu Ammonis* (CA) region CA1 (52% decrease), compared to the SO group (*p* < 0.0004). The treatment with NMP at the dose of 100 mg/kg resulted in values similar to those of the Pilo group (43% decrease) and, thus, did not significantly prevent the reduction in CA1 pyramidal neurons when compared to the SO group. In contrast, no statistical difference was noticed between the Pilo + NMP group treated with the dose of 200 mg/kg, and the SO group (Figure 3A).

In the CA3 region, a 69% reduction in viable cells (indicated by black arrows in Figure 3) was observed in the Pilo group, compared to the SO group. The treatment with NMP, at both doses, limited to 18% the decrease in the percentage of viable cells, in contrast to Pilo, which instead presented a significant lesion when compared to the SO group (*p* < 0.0001, one-way ANOVA followed by Dunnett’s test, Figure 3B). In the dentate gyrus (DG) area, there was no significant reduction of neurons in the granular layer, showing a greater resistance of these cells in this region (Figure 3C) as repeatedly reported in the literature on animal models of SE [29].

### 2.3. Ionized Calcium-Binding Adaptor Molecule 1 (Iba-1) Western Blot

**Figure 4:**
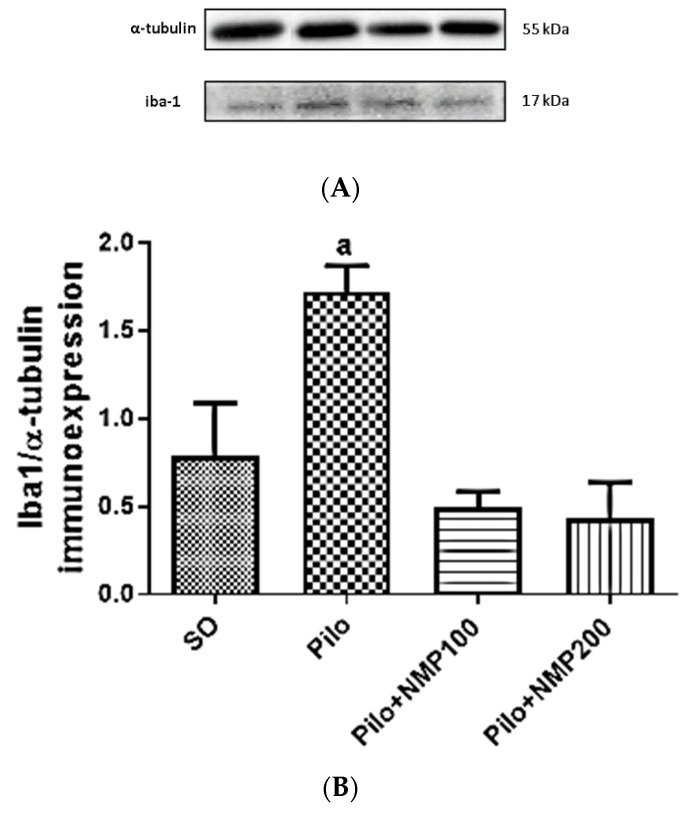
The *N*-methyl-(2S,4R)-trans-4-hydroxy-l-proline-rich fraction (NMP) partly reversed the increased microglia activation, in mice subjected to the intracerebroventricular (icv) injection of pilocarpine (Pilo), compared with the SO group as evaluated by western blot for the ionized calcium-binding adaptor molecule 1 (Iba-1). Representative blots (**A**) show the higher intensity band which corresponds to standard α-tubulin (55 kDa), and the 15 kDa band which corresponds to the Iba-1 band (four mice per group). Below, the densitometric quantification of the bands (**B**), performed by the Image J software (NHI, USA), is shown. a. vs. SO, *p* < 0.0033 (one-way analysis of variance and Dunnett’s multiple comparisons test).

The western blot for Iba-1 in hippocampi, standardized for α-tubulin, showed a 2.2-fold significant increase of this marker of inflammatory cells in the Pilo group compared to the SO group (*p* < 0.0033, one-way ANOVA followed by Dunnett’s test) (Figure 4). Remarkably, in both the PILO-NMP100 and PILO-NMP200 groups, the values were below the basal level of the SO group, even though not significantly.

### 2.4. GFAP Immunofluorescence

**Figure 5:**
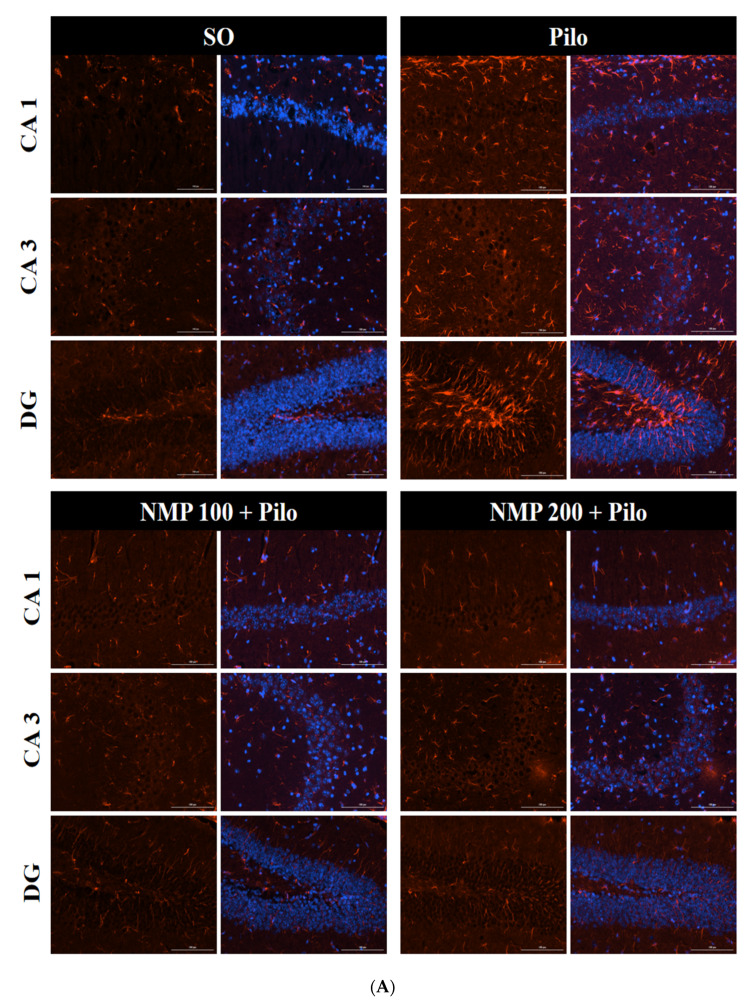

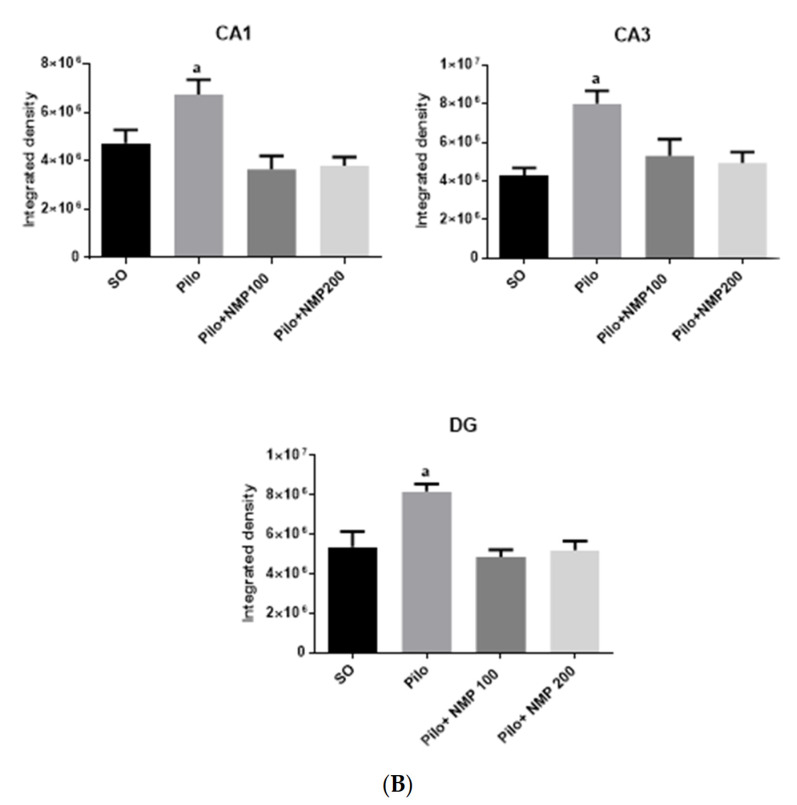
The *N*-methyl-(2S,4R)-trans-4-hydroxy-l-proline-rich fraction (NMP) completely prevented the increase in hippocampal glial fibrillary acidic protein (GFAP) immunoexpression in mice subjected to the intracerebroventricular (icv) injection of pilocarpine (Pilo). The animals received Pilo (300 µg/1 µL, icv) and, 24 h after, were treated with NMP (100 or 200 mg/kg, *per os*, *po*), for 15 days. The sham-operated (SO) group received the vehicle, distilled water, *po*. After the behavioral tests, the animals were euthanized for immunofluorescence assays, performed with four animals per group. On the left, photomicrographs are presented for each group, in hippocampal fields showing cells stained for GFAP only (in red) and nuclei counterstained with 4′,6-diamidino-2-phenylindole (DAPI) (in blue) (**A**). Quantification (bars on the right) is referred to the fluorescence integrated density in the various hippocampal subfields (**B**). CA1: a. vs. SO, *p* < 0.0002; CA3: a. vs. SO, *p* < 0.0001; DG: a. vs. SO, *p* < 0.0001; (one-way analysis of variance and Dunnett’s test for multiple comparisons).

The immunofluorescence analysis for GFAP (Figure 5A) showed in the CA1 a 1.5-fold significant increase of labeling in the Pilo group compared to SO (*p* < 0.0002, one-way ANOVA followed by Dunnett’s test). The immunolabelling intensity in both NMP100 and NMP200 groups was lower than the values found in the SO group (Figure 5B). Similar data were also seen in the CA3 and DG, in which a respective two-fold (*p* < 0.0001) and 1.8-fold (*p* < 0.0001) increase was present. Values were similar to those of the SO group in both NMP groups, with no significant difference among the three mentioned groups.

### 2.5. Target Prediction Results for NMP

To estimate the most probable human targets for NMP protective effects, we submitted the chemical structure of NMP (in SMILES format) to the SwissTargetPrediction server. The server algorithm predicted the most probable human proteins, based on the similarity of NMP with chemical structures of the drug database, and ranked proteins from the most to the less probable ones (Table 1). Based on this prediction, GAT1 was recognized as the most probable target for NMP, considering the quantitative probability rate and the number of known actives with similar chemical properties. According to these results, then we investigated the NMP interaction with GAT1, as well as its expression in our animals.

### 2.6. Molecular Docking for the NMP

**Figure 6:**
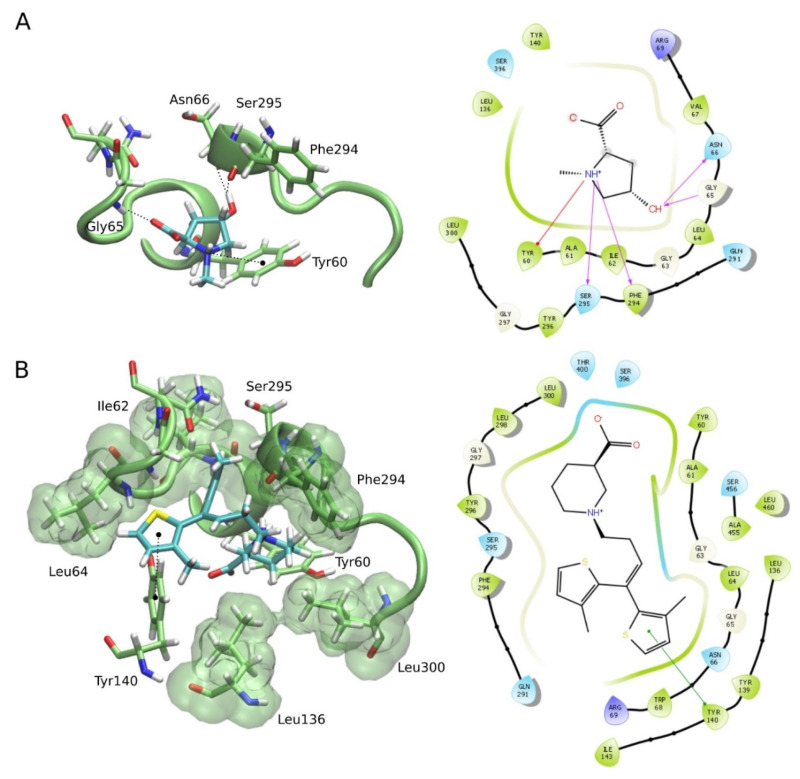
Predicted binding mode for the *N*-methyl-(2S,4R)-trans-4-hydroxy-l-proline-rich fraction (NMP) and the tiagabine molecule, into the GAT1 binding site. (**A**) Intermolecular 3D and 2D interactions of NMP with the main GAT1 model obtained by docking in the 3D representation; H-bonds are presented as black dashed lines and the hydrophobic interactions as green surfaces. In the 2D interaction diagram, hydrogen bonds are presented as magenta arrows and cation-Pi interaction as red arrows. (**B**) Intermolecular 3D and 2D interactions of tiagabine with GAT1: in the 3D representation, the Pi–Pi interaction is presented as black dashed lines and the hydrophobic interactions as a green surface. In the 2D interaction diagram, the Pi–Pi interaction is represented as green arrows.

Firstly, we built the human GAT1 structure through a homology modeling approach, using the SwissModel server. The model was based on dopamine receptor from Drosophila melanogaster (Protein Data Bank identifier 4XP4), which had 45.88% of sequence identity and 0.89% of coverage with the human GAT1. The model was analyzed at the MolProbity server and showed a MolProbity score of 1.35. The MolProbity score provides a log-weighted combination of the clashscore, the Ramachandran score not favored, and the percentage of bad side-chain rotamers, reflecting the crystallographic resolution at which those values would be expected. The analysis of the Ramachandran plot of the GAT1 model showed that 99.06% of the residues lie in the most favorable regions, which was more than satisfactory since ideally, 98% of the residues should lie in the favorable regions. After modeling, molecular docking calculations were performed in order to investigate the binding affinity of NMP against GAT1, as well as to study the possible binding mode and the interactions among them. NMP docked into GAT1 and presented a docking score of −4.38 kcal·mol^−^^1^. We also performed docking calculations of tiagabine, a GAT1 blocker that acts as a GABA reuptake inhibitor. Tiagabine docked into GAT1 and presented a docking score of −5.13 kcal·mol^−^^1^. Our data point out that the binding affinity and docking score of NMP against GAT1 was similar to that of tiagabine. However, tiagabine showed fewer hydrogen bonds with GAT1 residues and more hydrophobic interactions compared with NMP, which justify its slightly different docking score. Furthermore, the action mechanism of NMP is at least partly the result of its GAT1 blocking properties (Figure 6).

### 2.7. GAT1 Immunohistochemistry

**Figure 7:**
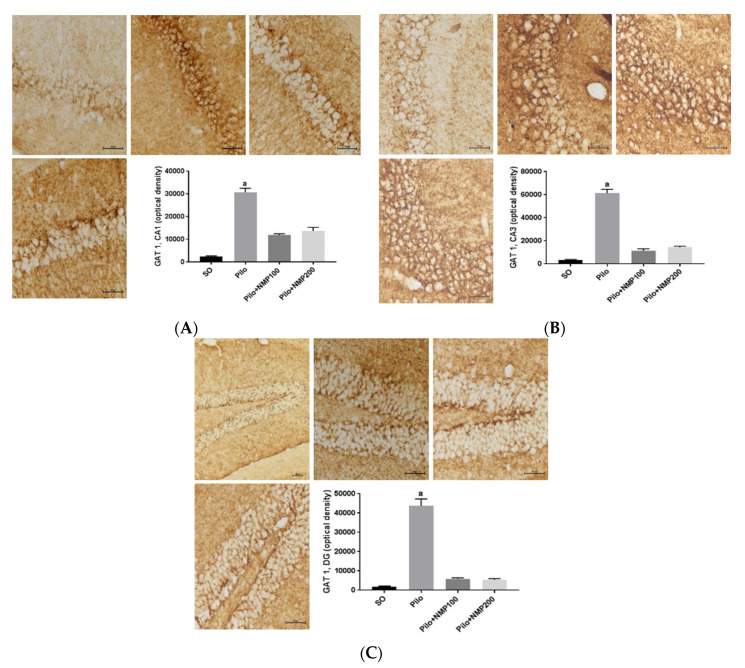
The *N*-methyl-(2S,4R)-trans-4-hydroxy-l-proline-rich fraction (NMP) prevented the hippocampal immunoexpression increases of the GABA transporter (GAT1), in the various hippocampal regions (**A**, *cornu Ammonis* 1—CA1; **B**, CA3; **C**, dentate gyrus—DG) of mice subjected to the intracerebroventricular (icv) injection of pilocarpine (Pilo), compared with the SO group. The animals received Pilo (300 µg/1 µL, icv) and, 24 h after, were treated with NMP (100 or 200 mg/kg, *per os*, *po*) for 15 days (Pilo + NMP100 and Pilo + NMP200 groups). The sham-operated (SO) group received distilled water (vehicle, *po*). After the behavioral tests, the animals were euthanized for the immunohistochemistry assays, performed in four animals per group. CA1: a. vs. SO, *p* < 0.0002. CA3: a. vs. SO, *p* < 0.0002. DG: a. vs. SO, *p* < 0.0002 (Kruskal–Wallis test and Dunn’s multiple comparisons test).

Immunohistochemistry findings for GAT1 were analyzed in the hippocampus. In the CA1 hippocampal subfield, GAT1 in the Pilo group was increased almost 12-fold (*p* < 0.0002, Kruskal–Wallis test followed by Dunn’s test) compared to the SO group. This change was much lower (respectively, 4.6-fold and 5.3-fold) in the Pilo + NMP groups at the doses of 100 and 200 mg/kg, and did not differ significantly from the levels of the SO group. In other words, NMP at both doses partly prevented the icv Pilo-induced GAT1 increase in CA1 (Figure 7A). A similar effect was seen in CA3, where GAT1 labeling was increased 17-fold (*p* < 0.0002), compared with the SO group. Although not significantly different from the SO group, fewer consistent (three-fold and four-fold) increases were seen after NMP treatment, respectively, at the doses 100 and 200 mg/kg (Figure 7B). Moreover, DG exhibited a remarkable 24-fold increase in GAT1 labeling in the Pilo group compared to the SO group (*p* < 0.0002). Although not significant, an increase (around three-fold) compared with the Pilo group was seen at both NMP doses (Figure 7C).

### 2.8. GAT1 Western Blot

**Figure 8:**
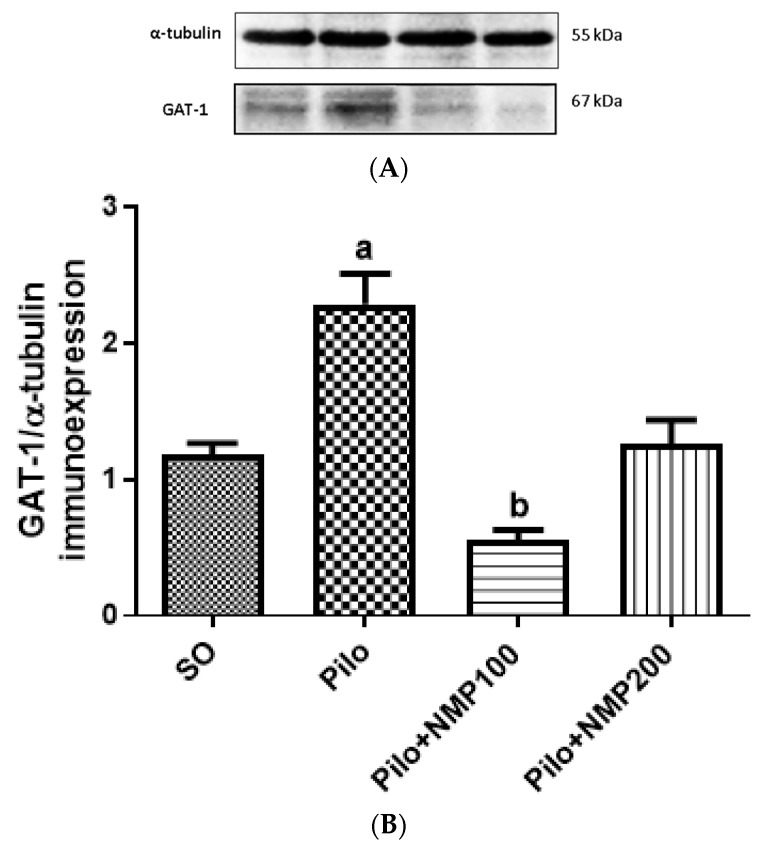
The *N*-methyl-(2S,4R)-trans-4-hydroxy-l-proline-rich fraction (NMP) partly prevented the increased γ-aminobutyric acid (GABA) transporter (GAT-1) expression in mice subjected to the intracerebroventricular (icv) injection of pilocarpine (Pilo), compared with the SO group as evaluated by western blot analysis. Representative blots (**A**) show the higher intensity band, which corresponds to standard α-tubulin (55 kDa), and the 67 kDa band corresponding to the GAT-1 band (four mice per group). Below, the densitometric quantification of the bands is shown (**B**), as performed by the ImageJ software (National Institute of Health - NHI, USA). a. vs. SO, *p* < 0.0014; b. vs. SO, *p* < 0.0293 (Kruskal–Wallis test and Dunn’s multiple comparisons test).

The results of the immunohistochemical analysis for GAT-1 were confirmed by western blot experiments in whole hippocampi. The Pilo group exhibited a 1.9-fold significant increase in GAT1 immunoexpression compared to the SO group (*p* < 0.0014, Kruskal–Wallis test followed by Dunn’s test). The group Pilo + NMP100 exhibited a GAT1 expression 53% lower compared to the Pilo group (*p* < 0.0293). There was no significant difference between the SO and Pilo + NMP200 groups (Figure 8).

## 3. Discussion

We showed that the NMP fraction from *Sideroxylon obtusifolium* leaves exerts anti-inflammatory and neuroprotective activities in the icv Pilo model of MTLE. The antiepileptic activity was demonstrated previously by our group, in the systemic Pilo model [30], but also in the model of icv Pilo, which was the subject of the present study. Most importantly, this effect was presented not only by NMP but also by l-proline and trans-hydroxyproline, suggesting that the NMP activity in the Pilo model of the mesial temporal lobe injury is due to its L-proline content. In the present study, by using the icv injection of Pilo, we showed that NMP exerts a protectant, presumably antiepileptic activity that has a relationship with its anti-inflammatory action, as we have demonstrated previously [28]. The icv injection of Pilo is an MTLE model, useful as a tool for investigating the mechanisms underlying the generation and maintenance of seizures [31].

Comorbid behavioral disorders are part of the epilepsy-interictal spectrum [32]. Epilepsy-related behavioral disorders include depression, psychosis, and severe cognitive deficits, which significantly impair a patient’s life functionality [33]. We showed that icv Pilo-treated mice presented a marked deficit in the short-term memory, which was prevented by both the NMP doses evaluated here. The ORT is a behavioral test often used for the investigation of several aspects of learning and memory in mice [34]. Pilo also significantly reduced the number of correct spontaneous alternations, compared with the SO group, as evaluated by the Y-maze test used to assess the working memory in mice [35]. Therefore, NMP, as other proline derivatives [36], can represent not only a useful tool for the control of the pro-seizure activity but also to counteract the epilepsy-associated cognitive deficits that represent a great burden for these patients.

According to Furtado and colleagues [37,38], icv Pilo in mice provokes remarkable neurodegeneration in CA1 and CA3 hippocampal subfields, as confirmed in the present study. Comparatively, kainic acid (after icv, as well as after intrahippocampal injections in Sprague-Dawley rats), which is a more widely used model of MTLE, induces neurodegeneration in the CA3, leaving the CA1 and DG almost intact [39,40]. Both NMP doses were able to protect the CA3 subfield, whereas only the highest dose was effective in the CA1 subfield. This observation suggests that the results obtained in the described behavioral tests could be more dependent on protection in the hippocampal CA3 subfield.

Epilepsy is a neurologic disease of sporadic and progressive disruption of neuronal activity or of a heterogeneous neuronal-glia network [41]. Evidence shows that brain activity and connectivity are drastically altered during epileptic seizures. Brain networks are known to shift from a balanced resting state to a hyperactive and hypersynchronous state [42,43]. Pilo-induced SE was shown to induce a prominent activation of astrocytes and microglia in the DG, from three up to 20 days after the initial seizures in rats [44]. The time interval of our study was within the mentioned period of glial activation and demonstrated that NMP is able to maintain both astrocytes and microglia in the resting state.

Microglia are known to play a critical role in brain homeostasis. Microglia are the brain resident immune cells that play important roles in the development and maintenance of neural circuits, and activated microglia exert different effects on brain function [45]. Recently [46], elevated mTOR signaling in mouse microglia was demonstrated to lead to phenotypic changes, without significant induction of pro-inflammatory cytokines, disrupting the brain homeostasis, and leading to reduced synaptic density, marked neuronal degeneration and massive proliferation of astrocytes. According to our findings, NMP at both tested doses has the potential to prevent all these detrimental effects.

In the present work, we showed that astrocytes were activated in the hippocampi of mice subjected to the icv injection of Pilo. These effects were substantially decreased after treatments of mice with NMP, at both doses, suggesting that NMP exerts a neuroprotective effect on epileptic mice. Astrocyte and microglial activation occur following seizures and, for this reason, could play an important role in epileptogenesis [47]. Importantly, these hippocampal changes manifested as increased Iba-1 and GFAP expressions occur rapidly after Pilo-induced seizures in rats, and last for several weeks [44]. An increase in Iba-1 positive cells was also demonstrated in the hippocampus after Pilo-induced SE, suggesting that SE triggers time-dependent alterations in the microglia morphology [48]. Although we did not investigate the morphological changes of microglia, the findings on Iba-1 expression in the presence of NMP administration suggest a possible effective modulation of the tested active compound.

The extensions of astrocytes with presynaptic terminals and postsynaptic spines generate a complex, so-called tripartite synapse [49]. The phenomenon named reactive astrogliosis is characterized by hypertrophy of primary processes and a dramatic increase in the expression of GFAP [17,50]. A number of studies have provided compelling evidence of dramatic alterations in the morphology and function of astrocytes in epilepsy, leading to reactive astrogliosis [51,52,53,54]. All these findings suggest that astrocytes are crucial players in epilepsy, thus representing an important target for the NMP effects.

In the last decades, computational methods have been widely used in drug development studies for providing several advantages compared to traditional methods. For example, computational tools brought to drug development pipelines higher successful rates, shorter time, and less costs. Also, these methods represent an ethical gain since they reduce the number of animals and biological resources used in experimental research [55]. Here, we firstly applied a target prediction algorithm to obtain the most probable biological targets for NMP interaction, which could guide our research efforts. Interestingly, we found for NMP several targets involved in the GABAergic transmission, being GAT1 the most predicted. Based on this, we conducted a molecular docking approach to better clarify the binding mode and affinity of NMP to this transporter. Our experiments showed docking score values of NMP that were quite similar to those of tiagabine, a GABA reuptake inhibitor. Tiagabine, an antiepileptic drug, has a specific and unique mechanism of action involving the inhibition of GABA reuptake into neurons and glia [56]. Despite facilitating the induction of GABA-mediated depolarization, tiagabine also increases the effectiveness of synaptic inhibition during the synchronous high-frequency activation of inhibitory interneurons [57]. By elevating GABA levels and availability in the synaptic cleft, GAT-1 inhibition represents an established approach for the treatment of epilepsy and is the potential target for the NMP mechanism of action.

However, other possible mechanisms might contribute to NMP effects. Indeed, midazolam, which is known to modulate the GABA_A_ receptor by interacting with the benzodiazepine binding site, was reported to modify potassium currents [58,59]. Moreover, vigabatrin has been shown to reduce astroglial the weak inward rectifier K^+^ (TWIK)-related acid-sensitive K^+^ channel 1 (TASK1) in the hippocampus of seizure-sensitive gerbils [60]. Recently, vigabatrin was also found to influence the activity of intermediate-conductance calcium-activated K^+^ channels in human glioma cells [61]. Another possible target of NMP could be the A_3_ purinoceptor, as revealed by our prediction analysis. This is a G_i/o_ protein-coupled receptor activated just at very high adenosine concentrations (i.e., during seizures or ischemia). The role of A_3_ in epilepsy and ictogenesis is poorly understood and does not appear to exert an univocal proconvulsant effect. Its activation has been demonstrated to elicit desensitization of GABA_A_ and A_1_ (anticonvulsant) adenosine receptors, as well as to modulate the presynaptic glutamate release and post-synaptic K^+^ currents, which are believed to be the A_3_ downstream effector mechanisms [62]. Thus, NMP effects could not be simply attributable to inhibition of GAT1 and the reduction in GABA clearance, because it might also be associated with potential modulatory effects on different types of ionic currents present in central neurons, astrocytes, or microglial cells, which would be intriguingly investigated in the near future.

## 4. Materials and Methods

### 4.1. Plant Material Extraction and Purification

*Sideroxylon obtusifolium* leaves were collected from specimens in the rural district from the Mauriti municipality, Ceará State, in August 2014. A voucher specimen (#10,648) was deposed in the Herbarium “Dárdano de Andrade Lima”, from the Regional University of Cariri (URCA), Crato-Ceará, Brazil, Dr. Maria Arlene Pessoa da Silva being the person responsible. The bioactive, NMP-enriched fraction was obtained by the procedure previously described [28], and dissolved in fresh distilled water (vehicle) before *po* administration. The quantitative ^1^H nuclear magnetic resonance (qHNMR) method was used to determine the *N-*methyl-trans-4-hydroxy-l-proline concentration in the methanol fraction (NMP) used in the present study. The quantification was based on the internal standard method, which is widely accepted as the primary approach to qNMR. The NMP content observed in the methanol fraction was 121.7 ± 5.1% mg/g, corresponding to 12% of NMP present in the leaves from *Sideroxylon obtusifolium*. Consistently, the ^1^H NMR spectra of NMP was the same as that of the isolated pure compound *N*-methyl-(2S,4R)-trans-4-hydroxy-l-proline.

### 4.2. Animals

Male Swiss mice (25–33 g; 8–9 per group before Pilo treatment, eight per group after SE) were kept at 25 ± 2 °C, under a 12/12 h light/dark cycle beginning at 7:00 a.m. Food and water were provided *ad libitum*. The experiments were carried out in strict observance of the USA National Research Council guidelines for the care and use of laboratory animals [63]. This study was submitted to and approved (2017, project code 59/17) by the Animal Research Ethical Committee of the Federal University of Ceará (CEUA/UFC).

### 4.3. Drugs and Chemicals

Pilo (Sigma-Aldrich, São Paulo, Brazil) was dissolved in artificial cerebrospinal fluid (aCSF) (composition in mM: NaCl 125, KCl 2.5, NaH_2_PO_4_ 5, CaCl_2_ 1.2, MgCl_2_ 1) [64], pH adjusted to 7.4. Ketamine and xylazine were from König (Argentina). Drug solutions were prepared freshly before use. All other drugs and reagents were of analytical grade. The solutions were administered in a volume of 0.1 mL/10 g of body weight.

### 4.4. Stereotaxic-Guided Injection of Pilo or aCSF in the Left Lateral Ventricle of the Brain, and Treatments

Mice were given intraperitoneal (ip) methyl scopolamine 3 mg/kg as prophylaxis for the peripheral effects of muscarinic activation by Pilo. Five min later, they were anesthetized with ketamine *plus* xylazine, respectively, 90 and 10 mg/kg ip. By using an electric razor, the fur was removed from the head. Then, the animals were placed and centered in a 10 μm precision motorized stereotaxic apparatus (51730M, Stoelting, Wood Dale, IL, USA), taking care to avoid ear bar misplacement. The eyes were kept wet by 0.9% saline to prevent keratitis. The skin disinfection was carried out by applying iodine-polyvinylpyrrolidone (Povidone, Vick Pharma, São Paulo, Brazil). Then, a midline incision between ears and eyes, about 1.5 cm long, was made with a scalpel. Validation of the flat skull position of reference was carried out by measuring and noticing bregma and lambda dorsoventral coordinates. From this point, the level of the tooth bar was lowered or lifted, so tilting the cranium downwards or upwards to measure the dorsoventral coordinates of bregma and lambda in a cycle repeated as many times as necessary until the dorsoventral coordinates of both craniometric points differ from each other not more than 10 μm. After that, taking as reference the mediolateral and craniocaudal coordinates of bregma, a point 0.5 mm caudal and 1.1 mm left-sided lateral to bregma was pinpointed on the skull cap corresponding to the projection of the left lateral ventricle of the brain [65]. A hole was drilled by a 1016 spherical FG dental bur mounted on a low rotation handpiece from Beltec, LB100, by a high speed friction grip adapter mandrel, taking care not to injure the dura and the underlying cortex. Eventual bleeding was plugged at this point. Then, the tip of the needle of the Hamilton syringe was placed at the center of the trepanation. Starting from the dorsoventral level of the skull surface at that point, the tip of the needle was dorsoventrally lowered 2.8 mm. Either Pilo 300 μg/1 μL diluted in aCSF, or aCSF (1 μL) were injected slowly (over 1 min). The needle was kept in place for 5 min to prevent reflux through the needle track. After that, the syringe was removed, and the wound edges were fitted together by nylon 4–0 thread in separate stitches [66]. Animals were left to recover from anesthesia on a thermal pad to avoid hypothermia, with the cranial part of the body slightly lifted to avoiding airway obstruction. They were injected twice a day for two days, in alternate times, with Ringer lactate solution and glucose 5% subcutaneously, approximately 0.5 mL/25 g of body weight. Animals were given distilled water, NMP 100, or 200 mg/kg (groups: SO, Pilo, Pilo + NMP100, and Pilo + NMP200 respectively) daily, over 15 days beginning from the first postoperative day. In this so-called “silent period”, between the primary injury and the clinical manifestation of disease, the epileptogenic process which renders the animals epileptic is thought to take place [67].

### 4.5. Behavioral Tests Performed on the 15th Postoperative Day

#### 4.5.1. Novel ORT

The novel ORT is used to evaluate recognition memory, that is, short term memory. This task is based on the innate tendency of rodents to explore unfamiliar objects within their environment. This test assesses the mouse’s ability to discriminate between familiar and novel objects. Firstly, mice were individually habituated to an open field plexiglass box (30 × 30 × 40 cm) for 5 min. After 15 min, mice were allowed to explore a set of two identical objects for 5 min (acquisition phase). These objects were suitably heavy and long to guarantee that mice could neither move them nor climb over them. After a 5 min interval, mice were presented to a similar set of objects in the same environment, with the replacement of one familiar object by a novel/unknown object (testing phase). The animals were allowed to freely explore the objects again for a 5 min period. A discrimination index was calculated as follows: (time exploring new object-time exploring familiar object)/(time exploring new object + time exploring familiar object) [68].

#### 4.5.2. Y-Maze Test

The working memory and learning were assessed by the rate of spontaneous alternations in the Y-maze three arms (40 × 5 × 16 cm), positioned at equal angles, as previously described [69]. Before running the test, the arms were numbered, and the animal was placed in one arm and spontaneously alternated the entries in the other arms for 8 min. The sequence of the arms into which the animal entered was then noted, and the information analyzed, in order to determine the number of arm entries without repetition.

### 4.6. Euthanasia and Tissue Harvesting

Immediately after behavioral tests, the animals were randomly assigned to groups for either immunohistochemistry or western blot assays. For immunohistochemistry, mice were deeply anesthetized with ketamine (90 mg/kg) and xylazine (15 mg/kg) ip. After they lost the withdrawal reflex to the hind paw pinch, the rib cage was cut longitudinally exposing the mediastinum. A cannula was inserted in the heart left ventricle. Then, the animals were transcardially perfused with 0.9% saline (about 60 mL) for vascular rinsing, and afterwards, with 60 mL of a 4% paraformaldehyde (PFA) solution in fosfate buffered saline (PBS) (pH 7.4). Skull was opened on an aluminum foil-covered ice bar, and brains were carefully collected, post-fixed for 2 h in the same PFA 4% solution at 4 °C, and then cryoprotected in 30% sucrose diluted in PBS (pH 7.4) for 72 h. Brains were sliced in a cryostat (Leica, Wetzlar, Germany) into 10 μm coronal sections, which were stored in cryoprotectant at −20 °C for further use. Diversely, for western blot assays, mice were euthanized by decapitation, brains were quickly dissected on an aluminum foil-covered ice bar, whole hippocampi were freshly harvested and stored in vials at −80 °C until use.

### 4.7. Nissl Staizning

In this study, cell viability was analyzed by the cresyl violet staining. The cresyl violet staining is used to highlight the Nissl corpuscles present in the cytoplasm of viable neurons [70]. The slides with the brain slices of cortex and hippocampus were dipped in distilled water for 1 min and immersed in a 0.5% cresyl violet solution prepared in acetate buffer (20% sodium acetate [2.7%] + 80% glacial acetic acid [1.2%]) for 3 min. The staining was desaturated with two washes in acetate buffer. Then, the sections were dehydrated in alcohol (50%, 70%, and 100%). Finally, they were dipped in xylol and assembled with Entellan (Merck, Whitehouse Station, NJ, USA). For the quantification of Nissl stained neurons, the slides were visualized with a microscope (Nikon Elipse E200, Nikon, Shinjuku, Japan) under a 100× magnification objective lens. Three slices of each animal were randomly selected, and the quantification of stained neurons was performed using the ImageJ software (NIH, Bethesda, MD, USA) with a grid of 1000 pixels in the hippocampus subfields CA1, CA3, and DG. The value of each animal was taken as the average of the three slices, and the results expressed as the number of viable cells. The cells were considered viable neurons when they presented violet staining in the cytoplasm, as well as normal morphological aspects (round or oval cells with centralized nuclei).

### 4.8. Drug Discovery Computational Tools

#### 4.8.1. Target Prediction

The prediction of biological targets of the NMP was performed through the SwissTargetPrediction Server [71]. This server estimates the most probable macromolecular targets of a small molecule, assumed as bioactive. The prediction is based on a combination of 2D and 3D similarity with a library of 370,000 known actives on more than 3000 proteins. The results are presented in probability rates that can guide the subsequent experiments.

#### 4.8.2. Homology Modeling and Molecular Docking Calculations: Comparing the Interaction of NMP and Tiagabine with GAT-1

The structure of sodium chloride dependent GAT1 was built by homology modeling, through the SwissModel server. The primary sequence of GAT1 was searched at the Uniprot server [71]. The model that presented higher sequence identity and coverage with the template was selected to the refinement step, at the GalaxyRefine server. The MolProbity server was used to add hydrogen atoms and to analyze the quality of the statistics of the modeled protein. The NMP, as well as the GAT-1 blocker tiagabine, were docked into GAT-1 using the Glide software in extra precision (XP) mode. We prepared the protein structures through a Protein Preparation Wizard tool, adding the hydrogen atoms and minimizing the energy, using the OPLS-2005 force field. The ligands were prepared through the LigPrep tool, correcting protonation, according to Epik, and performing energy minimization. The protein grid coordinates were built, based on the coordinates of the ligand cocaine, co-crystallized in the structure used as templates of the model (from PDB ID 4XP4). The Visual Molecular Dynamics program (VMD) [72,73] was used for the visual inspection of 3D docking poses and to render the 3D molecular images.

### 4.9. Immunohistochemistry for GAT-1

Sections operationally defined as −2.46 mm to −2.92 mm (caudal) to bregma containing hippocampi [60] were chosen and fixed in methanol. After cooling, they were washed four times with PBS, and the endogenous peroxidase blockade with a 3% H_2_O_2_ in PBS (15 min) was carried out. The sections were incubated overnight (4 °C) with the primary antibodies (anti-GAT-1 1:200, Abcam ab426, Cambridge, UK) in PBS, according to the manufacturer’s instructions. On the following day, the sections were washed in PBS four times, incubated (30 min) with the secondary biotinylated rabbit antibody (anti-IgG) in PBS (1:200), washed four times in PBS, and incubated (30 min) with the conjugated streptavidin-peroxidase complex (Burlingame, CA, USA). After rinsing, the sections were developed with 3,3-diaminobenzidine mounted on glass slides, dehydrated, and coverslipped for the analysis. The data were semi-quantified with the ImageJ software, NIH, USA [74].

### 4.10. Western Blotting Assays for GAT1 and Iba-1

Hippocampi for immunoblotting were washed with ice-cold PBS and put in radioimmunoprecipitation assay buffer (RIPA) lysis buffer (25 mM Tris—HCl, pH 7.6; 150 mM NaCl; 5 mM EDTA; 1% NP40; 1% Triton X-100; 1% sodium deoxycholate; 0.1% sodium dodecyl sulphate) with protease inhibitor (1 μL inhibitor: 100 μL RIPA). The lysed samples were transferred into microcentrifuge tubes, sonicated two times for 5 s, and then cleared by centrifugation (12,000 rpm, 15 min) at 4 °C. Total protein concentration in lysates was determined by the bicinchoninic acid (BCA) method according to the manufacturer’s protocol (Pierce BCA Protein Assay Kit, Thermo Fisher Scientific, Waltham, MA, USA). SDS polyacrylamide gel electrophoresis (10%) was performed using 20 μg of protein (previously prepared with Laemmli sample buffer and heated to 95 °C for 5 min). The proteins were transferred to PVDF membrane, blocked with 5% BSA for 1 h, and incubated overnight with rabbit polyclonal for Iba-1 (1:1000, Santa Cruz Biotechnology, Dallas, TX, USA) or rabbit polyclonal antibody for GAT-1 (1:600) or mouse antibody for α-tubulin IgG primary antibody (1:4000; Sigma-Aldrich). After washing, the blots were incubated with horseradish peroxidase-conjugated goat anti-rabbit IgG secondary antibody (1:1000, Thermo Fisher Scientific, Waltham, MA, USA) or goat anti-mouse IgG secondary antibody (1:1000, Thermo Scientific) for 90 min at room temperature. The signal was detected using the ECL system (Bio-RAD, Hercules, CA, USA) according to the manufacturer’s instructions, and then the bands were captured with a CCD camera using the ChemiDoc system (Bio-Rad, Hercules, CA, USA). Densitometric quantification of bands was performed with NIH ImageJ software (*n* = 4/group).

### 4.11. Immunofluorescence for GFAP

Slices containing hippocampi, identical to those aforementioned, were rinsed four times in PBS. They underwent an antigen recovery process and were incubated overnight at 4 °C with the mouse monoclonal anti-GFAP (1:200, Santa Cruz Biotechnology). After, slices were rinsed four times with PBS and then incubated for 2 h at room temperature with AlexaFluor-594 conjugated goat anti-mouse IgG antibody (1:400; Invitrogen, Carlsbad, CA, USA). Finally, they were stained with 1 μg/mL 4′,6-diamidino-2-phenylindole (DAPI) (Invitrogen, Carlsbad, CA, USA). Subsequently, slides were rinsed in PBS and coverslipped using ProlongGoldAntifadeMountant (ThermoFisher Scientific, Waltham, MA, USA). Slides were imaged using a Zeiss LSM 700 confocal microscope (Carl Zeiss, White Plains, NY, USA) through a magnification of 20 objective lens, at constant exposure, gain, and offset. The hippocampal subfields CA1, CA3, and DG were identified according to Paxinos, and Franklin’s Mouse Brain Atlas [65] and four to five photomicrographs of each area for each group were analyzed. The experimenter who took the images was blinded to treatments. The fluorescence intensity was semi-quantitative, using the ImageJ software package.

### 4.12. Statistical Analysis

Data were tested for goodness of fit to a normal distribution and are presented as mean ± SEM. The analysis was performed by one-way ANOVA, followed by the Dunnett’s for multiple comparisons test or the nonparametric rank-based Kruskal–Wallis test followed by the Dunn’s multiple comparisons test. Values of *p* < 0.05 were considered as significant.

## Figures and Tables

**Table 1:** Predicted targets for NMP based on its chemical similarity with SwissTargetPrediction drug database.

Target	Uniprot ID	Target Class	Probability	Known Actives (3D/2D)
GABA transporter 1	P30531	Electrochemical transporter	0.03	5/4
GABA A receptor lpha-3/beta-2/gamma-2	P34903 P47870 P18507	Ligand-gated ion channel	0.03	3/0
GABA A receptor alpha-2/beta-2/gamma-2	P47869 P47870 P18507	Ligand-gated ion channel	0.03	4/0
Renin	P00797	Protease	0.03	0/13
Egl nine homolog 1	Q9GZT9	Oxidoreductase	0.02	1/0
Adenosine A3 receptor	P0DMS8	Family A G protein-coupled receptor	0.02	2/3
GABA-B receptor	O75899 Q9UBS5	Family C G protein-coupled receptor	0	8/0
Thrombin	P00734	Protease	0	0/8
Leukotriene A4 hydrolase	P09960	Protease	0	0/4
GABA receptor rho-1 subunit	P24046	Ligand-gated ion channel	0	4/0
Phospholipase A2 group IIA	P14555	Enzyme	0	0/6
Phospholipase A2 group V	P39877	Enzyme	0	0/3
Integrin alpha-IIb/beta-3	P08514 P05106	Membrane receptor	0	0/11
Leucine aminopeptidase	P28838	Protease	0	0/5
Bile acid receptor FXR	Q96RI1	Nuclear receptor	0	0/1
Neprilysin	P08473	Protease	0	0/23
Voltage-gated calcium channel alpha2/delta subunit 1 (by homology)	P54289	Calcium channel auxiliary subunit alpha2delta family	0	10/0
Metastin receptor	Q969F8	Family A G protein-coupled receptor	0	0/2
11-beta-hydroxysteroid dehydrogenase 1	P28845	Enzyme	0	0/9
Nitric-oxide synthase, brain	P29475	Enzyme	0	0/9

## Abbreviations

aCSF	artificial cerebrospinal fluid
ANOVA	analysis of variance
CA	*cornu Ammonis*
DG	dentate gyrus, dentate fascia
GAT1/GAT-1	GABA transporter type 1, GABA-Na^+^ symporter 1
GFA	glial fibrillary acidic protein
Iba-1	Ionized calcium-binding adaptor molecule 1
icv	intracerebroventricular route of administration
MTLE	mesial temporal lobe epilepsy
NMP	*N*-methyl-(2S,4R)-trans-4-hydroxy-l-proline
ORT	novel object recognition test
Pilo	pilocarpine
PBS	phosphate buffered saline
